# 多发性骨髓瘤代谢组学研究进展

**DOI:** 10.3760/cma.j.cn121090-20241205-00536

**Published:** 2025-10

**Authors:** 烜 叶, 正旭 孙, 晓燕 屈, 磊 范

**Affiliations:** 江苏省人民医院（南京医科大学第一附属医院）血液科，南京 210029 Department of Hematology, the First Affiliated Hospital with Nanjing Medical University, Jiangsu Province Hospital, Nanjing 210029, China

## Abstract

多发性骨髓瘤（multiple myeloma，MM）是一种克隆性浆细胞异常增生的恶性疾病，代谢重编程是其重要特征。参与细胞功能的氨基酸、葡萄糖、脂质和核苷酸的异常代谢在MM的增殖、转移、免疫逃逸以及药物耐药等诸多关键病理过程中发挥重要作用。本文对利用代谢组学技术在分子水平上探寻MM的生物标志物以及针对代谢相关分子靶点的治疗手段进行综述，旨在为深入研究MM的代谢机制提供参考，推动MM临床诊疗的创新。

## Abstract (en)

Multiple myeloma（MM）is a malignant disease characterized by clonal plasma cell proliferation, with metabolic reprogramming. Abnormal metabolism of amino acids, glucose, lipids and nucleotides plays a crucial role in various pathological processes in MM, such as proliferation, metastasis, immune escaping and drug resistance. This review summarized the application of metabolomics technology in identifying biomarkers for MM and targeting related metabolic pathways. The objective is to guide further investigations into the metabolic mechanisms underlying MM and encourage innovation in clinical diagnosis and treatment strategies for this disease.

多发性骨髓瘤（multiple myeloma，MM）是血液系统第二常见的恶性肿瘤，其特征是骨髓中克隆性浆细胞的异常增殖[1]。近年来针对MM的治疗手段不断进步，但目前仍无法治愈，患者面临复发和耐药等挑战[2]。代谢组学能够全面分析生物体内小分子代谢物的变化，该技术被广泛应用于寻找MM病理生理过程中的差异代谢分子，作为疾病早期诊断及预后监测的生物学标志物，并为开发新的治疗策略提供潜在靶点。

本文旨在总结MM代谢组学领域的最新研究成果，探讨代谢异常在MM发生发展中的作用及其临床转化前景。随着代谢组学技术的不断进步，我们期待在MM的早期诊断、治疗监测和个体化治疗方面能够取得更多突破，为患者提供更有效的治疗方案。

一、代谢组学概述

代谢组学通过核磁共振波谱、气相色谱-质谱、液相色谱-质谱及毛细管电泳-质谱等检测方法，对生物体内小分子代谢物（相对分子质量为50～1 500）进行动态监测。代谢组学可分为靶向和非靶向两大研究策略：非靶向代谢组学通过无偏向性地检测样本全谱代谢物来筛选差异代谢物；靶向代谢组学则聚焦于特定的差异代谢物，主要应用于代谢通路的深度验证研究[3]。

肿瘤细胞通过重塑葡萄糖、脂质及氨基酸代谢网络，使自身在营养匮乏的肿瘤微环境（tumor microenvironment，TME）中维持持续增殖转移的潜能，这被称作肿瘤的代谢重编程[4]。其中，Warburg效应作为核心特征，表现为肿瘤细胞即使在富氧条件下，仍优先通过糖酵解途径将葡萄糖转化为乳酸，而非通过线粒体氧化磷酸化获取能量。此外，研究发现肿瘤细胞具有对电子受体和氮的需求增加、对氧化应激保护的依赖增加等代谢特征[5]。通过代谢组学技术解析特征性代谢通路，开发靶向代谢通路的新型治疗策略。

二、MM的代谢特征

（一）氨基酸代谢

1. 谷氨酰胺代谢增强：MM是唯一同时表现出“谷氨酰胺成瘾性”与“内源性合成缺陷”的恶性肿瘤，其增殖完全依赖外部谷氨酰胺供应[6]。大多数MM患者的浆细胞缺乏谷氨酰胺合成酶的表达，同时高表达谷氨酰胺酶（glutaminase，GLS）。与意义未明单克隆丙种球蛋白血症（monoclonal gammopathy of undetermined significance，MGUS）相比，MM患者的骨髓微环境中出现低谷氨酰胺和高谷氨酸的代谢失衡，从而促使间充质基质细胞表达谷氨酰胺合成酶，阻碍其成骨细胞分化，进而引起MM典型的溶骨性病变[7]。

2. 支链氨基酸代谢异常：MM患者体内对亮氨酸、异亮氨酸和缬氨酸等支链氨基酸（branched chain amino acid，BCAA）的利用率高，导致其血清中BCAA水平显著下降[8]–[9]。MM的进展同时涉及乳酸代谢重编程与BCAA代谢异常调控。在高危患者群体中，细胞毒性淋巴细胞数量呈现特征性衰减，这表明BCAA代谢产物可能减弱TME中免疫细胞功能。研究人员根据乳酸和BCAA的代谢特征，开发了一种MM的预后模型[10]。

3. 甲硫氨酸循环异常：甲硫氨酸循环是细胞内的重要代谢途径，参与多种生物分子合成及甲基化。肿瘤细胞的生长发育依赖于外源性甲硫氨酸，这种现象被称为Hoffman效应[11]。在MM患者的骨髓上清液中，甲硫氨酸和半胱氨酸水平显著升高，同时参与甲硫氨酸循环的酶在MM细胞中高表达，可以推测甲硫氨酸-半胱氨酸代谢轴的这些异常变化调控了蛋白质的甲基化修饰、核苷酸的合成和代谢等途径，最终进一步影响了MM的生物学行为和疾病进展[12]。此外，溶质载体家族38成员A1（solute carrier family 38 member A1，SLC38A1）介导的丝氨酸从骨髓液转运至巨核细胞，通过影响甲硫氨酸循环和S-腺苷甲硫氨酸依赖的甲基化反应来调节基因表达，进而抑制巨核细胞的分化，最终导致血小板生成减少，其与MM患者的预后不良密切相关[13]。

（二）葡萄糖代谢

在MM细胞中，糖酵解增强，其受到多种因素的调控。MM细胞中上调的葡萄糖转运蛋白家族成员（如GLUT4、GLUT8及GLUT11）增强了葡萄糖的跨膜转运，以满足肿瘤细胞的能量需求[14]。叉头框蛋白M1（forkhead box protein M1，FOXM1）作为MM代谢的重要调节因子，具有促进葡萄糖摄取、乳酸分泌及氧气消耗的作用，可同时影响糖酵解与氧化磷酸化能量代谢途径[15]。

（三）脂质代谢

1. 脂肪酸合成增加：MM细胞可以诱导骨髓脂肪细胞脂解，从而提高TME中脂肪酸水平。研究表明，在小鼠MM细胞与脂肪细胞共培养实验中，脂肪细胞的脂解活性与MM细胞的脂肪酸摄入量呈正相关[16]。此外，MM细胞还可能通过上调脂肪酸合成的关键酶即乙酰辅酶A羧化酶的表达，促进脂肪酸的从头合成[17]。同时，MM细胞通过脂肪酸转运蛋白（fatty acid transporters，FATP）1/4主动摄取脂肪酸，维持自身存活与增殖。

2. 脂肪酸氧化改变：脂肪酸氧化分解生成的乙酰辅酶A通过三羧酸循环和氧化磷酸化生成三磷酸腺苷（adenosine triphosphate，ATP），从而提供MM细胞增殖与侵袭所需的能量。在基础和应激条件下，MM细胞依赖长链脂肪酸β-氧化获取能量，同时CD28作为促生存/抗化疗的信号，激活Ca^2+^-腺苷酸活化蛋白激酶-Unc-51样自噬激活激酶1（Ca^2+^-AMPK-ULK1）轴，导致脂噬和脂肪酸氧化增加，从而维持MM细胞存活[18]。

3. 胆固醇代谢异常：胆固醇是细胞膜的主要成分，影响细胞增殖。研究表示，MM患者的总胆固醇、高密度脂蛋白胆固醇（high-density lipoprotein cholesterol，HDL-C）和低密度脂蛋白胆固醇均显著降低。载脂蛋白水平的降低不仅与MGUS向MM的转化进程相关[19]，还与MM疾病进展风险有关[20]。

（四）核苷酸代谢

肿瘤细胞需要增加核苷酸的合成，为DNA复制和RNA合成提供必要的原料。研究发现，嘧啶合成的关键酶即二氢乳清酸脱氢酶（dihydroorotate dehydrogenase，DHODH）在MM细胞中异常表达[21]，促使MM细胞获取更多的嘧啶核苷酸用于DNA和RNA的合成。

三、MM诊疗靶点探索

现有的代谢组学研究利用相关技术对MM患者的体液样本进行分析，筛选出与MM诊断及预后相关的代谢标志物，为MM危险度分层以及治疗靶点提供新的方向。

（一）氨基酸代谢诊疗靶点

在MM进展过程中，骨髓微环境中异常增加的氨基酸是MM细胞的重要营养来源。下调溶质载体家族6成员9（solute carrier family 6 member 9，SLC6A9）可阻断MM细胞摄取骨髓中因骨胶原降解而增多的甘氨酸，从而引起细胞内谷胱甘肽（glutathione，GSH）水平下降和DNA损伤增加，进而导致细胞周期阻滞[22]。然而，有研究在泊马度胺耐药的MM患者中发现外源性补充甘氨酸可以逆转这种耐药性[23]。同样，限制外源性摄入氨基酸也可能有治疗效果。例如，低苯丙氨酸饮食可作为MM辅助治疗手段，苯丙氨酸的缺失通过激活转录因子3-增强子结合蛋白同源蛋白-死亡受体5（ATF3-CHOP-DR5）信号轴扰乱内质网稳态，导致MM细胞凋亡[24]。

氨基酸代谢与表观遗传修饰紧密相关，通过改变DNA和组蛋白的修饰，影响肿瘤的发生发展[25]。谷氨酰胺的分解可以增加MM细胞的活力，作为该途径的关键酶，GLS的抑制剂CB-839能够增强组蛋白去乙酰化酶抑制剂的作用，同时提高染色质特定区域的组蛋白乙酰化水平，这些变化调控细胞凋亡相关蛋白的表达和稳定性，最终诱导细胞凋亡[26]。果蝇Zeste基因增强子人同源物2（enhancer of Zeste homolog 2，EZH2）是一种催化组蛋白H3第27位赖氨酸（H3K27me3）甲基化的甲基转移酶，抑制EZH2活性可显著降低多种MM细胞系的体内外存活率，其中机制涉及EZH2抑制剂调控甲硫氨酸循环相关基因的miRNA，从而诱导同型半胱氨酸积累和DNA损伤，最终导致G2期阻滞及细胞凋亡[27]。此外，血浆中的氨基酸也可用于MM的诊断，其中天冬氨酸和苏氨酸水平可能成为诊断及风险预测的重要代谢标志物[28]–[29]。

氨基酸的摄取和代谢变化可能引起MM治疗相关不良反应。卡非佐米对芳香族氨基酸的代谢途径有显著影响，这些代谢物可能成为卡非佐米相关肾不良反应的潜在生物标志物[30]。牛磺酸去氧胆酸可能是卡非佐米心血管不良事件的潜在生物标志物，研究者构建了相关代谢物的风险评分，用于评估发生心血管不良事件的风险[31]。

综上，氨基酸代谢产物天冬氨酸、苏氨酸和芳香族氨基酸，以及相关分子SLC6A9、SLC38A1、GLS、EZH2在MM的诊疗中显示出潜在价值。

（二）糖代谢诊疗靶点

在缺氧的TME中，低氧诱导因子1（hypoxia-inducible factor 1，HIF-1）会显著上调糖酵解途径中代谢酶的活性及表达，其中，葡萄糖代谢的关键酶如GLUT[32]、己糖激酶2（hexokinase-2，HK2）[33]、磷酸果糖激酶[34]、丙酮酸激酶同工酶2[35]、乳酸脱氢酶[36]等均为肿瘤治疗潜在靶点。针对这些关键酶的抑制剂（如GLUT4抑制剂、PFK158和5MPN）能够抑制肿瘤的生长。当糖酵解速率超过线粒体还原型烟酰胺腺嘌呤二核苷酸（NADH）穿梭体的最大转运能力时，增殖细胞主要将葡萄糖转化为乳酸[37]。过量乳酸会抑制T细胞功能，导致免疫治疗的抗肿瘤效果不佳[38]。Chong等[39]通过代谢组学分析发现，蛋白激酶C α（protein kinase C α，PKCα）在t（4;14）的MM细胞中过表达并通过磷脂酰肌醇3激酶-蛋白激酶B（PI3K-Akt）信号通路调控HK表达及活性，从而导致乳酸增多。此外PKCα以非依赖cereblon蛋白的方式诱导免疫调节剂耐药，提示乳酸可作为疗效监测的生物标志物。

葡萄糖-6-磷酸脱氢酶（glucose-6-phosphate dehydrogenase，G6PD）是磷酸戊糖途径（pentose phosphate pathway，PPP）的限速酶，肿瘤细胞中上调的G6PD会产生过量烟酰胺腺嘌呤二核苷酸磷酸（NADP），从而对表皮生长因子受体（epidermal growth factor receptor，EGFR）抑制剂耐药，将EGFR抑制剂与PPP抑制剂6AN联合使用，可在KRAS/NRAS/BRAF野生型MM细胞中产生协同的细胞毒性作用[40]。通过非靶向代谢组学，研究者发现MM患者血清中，脱氢表雄酮硫酸酯（G6PD抑制剂的前体）表达下降会引起G6PD升高，进而激活酶促PPP途径和非酶促Wnt/β-连环蛋白（Wnt/β-catenin）信号通路，诱导MM细胞产生耐药性[41]。

有氧条件下，糖代谢生成的丙酮酸进入线粒体参与三羧酸循环，并通过氧化磷酸化为细胞生长供能。氧化磷酸化依赖于线粒体呼吸链，因此，破坏线粒体呼吸链结构成为MM潜在的治疗靶点。Thongon等[42]的研究表明，对反义寡核苷酸耐药的MM细胞，在DNA损伤激活后，会通过激活氧化磷酸化启动代谢重编程维持生存，而抑制线粒体DNA修复蛋白DNA2可诱导线粒体呼吸链结构紊乱，促使耐药细胞凋亡，提示DNA2可作为克服DNA损伤治疗抵抗的靶点。热休克蛋白60（heat shock protein，HSP60）下调也可以破坏MM细胞线粒体呼吸链完整性，引起葡萄糖代谢与肌酸代谢紊乱，提示HSP60可成为MM治疗的新靶点[43]。FOXM1正向调控氧化磷酸化，其抑制剂NB73通过增强蛋白酶体介导FOXM1降解，从而治疗MM，并与HSP90具有协同作用[15]，然而，目前尚未明确二者是否通过干扰线粒体呼吸链结构削弱氧化磷酸化。

综上，MM通过上调糖代谢途径中的一些代谢酶来维持细胞生长。此外，相关蛋白（如DNA2、FOXM1、HSP60）也促进肿瘤细胞增殖，这些代谢分子可作为MM治疗的新靶点。

（三）脂质代谢诊疗靶点

游离脂肪酸对MM细胞具有浓度依赖性效应：低浓度促进增殖，高浓度诱导脂肪毒性，因此限制脂肪酸摄取是一种潜在的治疗策略。在小鼠浆细胞瘤模型中，外源性花生四烯酸的治疗可降低肿瘤负荷[16]。脂肪酸可以逆转耐药，Chen等[44]发现ω-3脂肪酸二十二碳六烯酸（docosahexaenoic acid，DHA）/二十碳五烯酸（eicosapentaenoic acid，EPA）通过激活核因子E2相关因子2-活化转录因子3/4-谷胱甘肽特异性γ谷氨酰环转移酶1（NRF2-ATF3/4-CHAC1）信号通路促进GSH降解，通过降低GSH水平增强硼替佐米的细胞毒性，从而克服MM硼替佐米耐药。脂质是细胞膜的重要组成部分，Besse等[45]通过代谢组学证实了奈非那韦通过改变细胞膜脂质组成及流动性，调控膜相关蛋白功能，进而发挥抗癌作用。以奈非那韦为基础的治疗可使65%的蛋白酶体抑制剂难治性晚期MM患者获得缓解或疾病稳定[46]。

脂质代谢的差异可以帮助临床制定生物标志物。一项基于32例骨髓瘤患者的血清和尿液样本的核磁共振波谱代谢组学研究发现，肉碱和乙酰肉碱可作为疾病诊断及复发监测的潜在标志物[47]。有研究在比较了伴或不伴髓外病变的MM患者血浆的代谢特征后，发现己糖神经酰胺（d18:1/20:0）、甘油三酯（16:0_34:2）、甘油三酯（22:4_32:0）、甘油三酯（18:2_32:0）和牛磺酸具有髓外病变诊断潜力[48]。低水平HDL-C与血液系统恶性肿瘤风险增加显著相关，提示HDL-C可能是MM的独立危险因素和临床前标志物[49]。进一步研究证实HDL水平<28 mg/dl患者的无进展生存期显著缩短[50]。

综上，代谢组学不仅筛选出肉碱、乙酰肉碱、HDL-C等标志物，还揭示FATP 1/4、DHA/EPA等治疗靶点，为MM诊疗提供新视角。

（四）核苷酸代谢诊疗靶点

嘧啶合成途径可作为MM诊治的潜在分子靶点。Bardeleben等[51]通过代谢组学筛选发现，一磷酸腺苷类似物AICAr能够选择性调控核苷酸代谢，通过抑制尿嘧啶核苷酸（uracil nucleotide，UMP）合成相关酶的活性，阻断嘧啶从头合成。DHODH抑制剂通过干扰嘧啶的合成，诱导肿瘤细胞S期阻滞并下调c-Myc蛋白表达，进而抑制MM细胞的生长[52]。

嘌呤合成途径可能与MM耐药机制密切相关。Koomen等[53]报道了美法仑耐药的MM细胞的嘌呤代谢物水平发生显著变化，嘌呤合成及其补救途径相关酶活性上调，促进DNA合成与修复，从而抵抗美法仑引起的DNA损伤，这提示抑制嘌呤合成途径关键酶可能改善美法仑疗效。此外，MM患者血清中嘌呤代谢产物尿酸水平为健康志愿者的9.39倍，可作为诊断及预后评估的新型生物标志物[54]。

综上，靶向AICAr、DHODH等分子及监测核苷酸代谢相关代谢物，在MM诊疗中具有潜在应用价值。

四、总结与展望

代谢组学研究揭示了MM特征性代谢通路，但研究仍处于起步阶段，还面临许多问题和挑战，如代谢标志物的特异性和敏感性有待提高以及多组学技术整合深度有限等。未来随着代谢组学技术迭代及多组学整合应用的推进，MM临床诊疗有望迎来新突破。
